# Supplementary material for: MetS Prevalence and Its Association with Dietary Patterns among Chinese Middle-Aged and Elderly Population: Results from a National Cross-Sectional Study
Source: Nutrients. 2022 Dec 13;14(24):5301. doi: 10.3390/nu14245301 (PMC9788494; doi:10.3390/nu14245301)
Supplement: Supplementary file 1 [file nutrients-14-05301-s001.zip › nutrients-2051676-supplementary.pdf]

Supplementary Materials

Table S1. Food categories for dietary pattern in the study.

No.	Food categories	Foods name
1	Rice products	
2	Wheat products	
3	Other cereals	oat, sorghum, maize, millets, buckwheat, etc
4	Tubers	taro, sweet potatoes, Chinese yam, potatoes, etc.
5	Dry legume	peas, red beans, mung beans, kidney beans, cowpea, etc.
6	Legume products	green beans, soybeans, black beans, bean curd, tofu, soybean milk, etc.
7	Dark colored vegetables	spinach, broccoli, tomato, carrot, chili peppers, purple cabbage, lettuce, etc.
8	Light colored vegetables	cucumber, eggplant, celery, zucchini, lotus root, etc.
9	Fungi and algae	edible mushroom, kelp, laver
10	Fresh fruits	apple, kiwi fruit, blueberry, banana, grape, watermelon, etc.
11	Nuts	walnuts, chestnuts, peanuts,
12	Pork	
13	Other red meat	beef, mutton, horsemeat, etc.
14	Red meat offal	liver, belly, lung, etc.
15	Poultry	chicken, duck, goose, etc.
16	Milk and dairy products	Pure milk, milk powder, cheese, yogurt
17	Eggs	
18	Aquatic products	fish, shrimp and shellfish
19	Cakes and desserts	cakes, chocolate, cookie, pasty, etc.
20	Candy and starch	white sugar, brown sugar, vermicelli, etc.
21	Vegetable oil	soybean oil, rapeseed oil, salad oil, sunflower seeds oil, etc.
22	Animal oil	lard oil, beef tallow, etc
23	Salt	
